# Strategies of Mesenchymal Invasion of Patient-derived Brain Tumors: Microenvironmental Adaptation

**DOI:** 10.1038/srep24912

**Published:** 2016-04-25

**Authors:** Junghwa Cha, Seok-Gu Kang, Pilnam Kim

**Affiliations:** 1 Department of Bio and Brain Engineering, KAIST, Daejeon 34141, Korea; 2 Department of Neurosurgery, Severance Hospital, Yonsei University College of Medicine, Seoul 03722, Korea

## Abstract

The high mortality in glioblastoma multiforme (GBM) patients is primarily caused by extensive infiltration into adjacent tissue and subsequent rapid recurrence. There are no clear therapeutic strategies that target the infiltrative subpopulation of GBM mass. Using mesenchymal mode of invasion, the GBM is known to widely infiltrate by interacting with various unique components within brain microenvironment such as hyaluronic acid (HA)-rich matrix and white matter tracts. However, it is unclear how these GBM microenvironments influence the strategies of mesenchymal invasion. We hypothesize that GBM has different strategies to facilitate such invasion through adaptation to their local microenvironment. Using our *in vitro* biomimetic microenvironment platform for three-dimensional GBM tumorspheres (TSs), we found that the strategies of GBM invasion were predominantly regulated by the HA-rich ECM microenvironment, showing marked phenotypic changes in the presence of HA, which were mainly mediated by HA synthase (HAS). Interestingly, after inhibition of the *HAS* gene, GBM switched their invasion strategies to a focal adhesion (FA)-mediated invasion. These results demonstrate that the microenvironmental adaptation allowed a flexible invasion strategy for GBM. Using our model, we suggest a new inhibitory pathway for targeting infiltrative GBM and propose an importance of multi-target therapy for GBM, which underwent microenvironmental adaptation.

Invasion ability toward surrounding tissue in the progression of malignant tumors is a main cause for metastasis and relapse^1^. Tumor cell invasion is predominantly regulated by distinctive biophysical and biochemical stimuli originating from the complex network of extracellular matrix (ECM) in their microenvironment^2^,^3^. During tumor progression, dynamic ECM remodeling mediated by reciprocal interactions between the ECM and tumor cells leads to facilitate tumor invasion^3^. Understanding the mechanisms of interactions between infiltrative tumor cells and ECM microenvironment is essential for developing new therapeutic inventions for malignant tumors.

Tumor cells possess a broad pattern of invasion mechanism, such as individual and collective cell migration, in response to different conditions, thus efforts to understand how ECM composition, mechanical properties (e.g., stiffness or porosity), and topography influence on the mechanisms of tumor invasion have been ongoing for decades^4^,^5^,^6^,^7^,^8^,^9^,^10^,^11^.

Unlike other systemic tumor, malignant brain tumors, especially glioblastoma multiforme (GBM), exhibit unique features of invasion. GBM invades locally inside brain (micro-metastasis) and spreads in a single-cell level, rather than metastasizes to distant organs. Moreover, GBM cells barely penetrate the subarachnoid space or intravasate into the cerebral microvasculature^12^,^13^. Although GBM invasion is confined within the intracranial spaces, patients have poor prognosis. The pro-invasive microenvironment for GBM remains unclarified, as there are no plausible models that reflect the composition and structural elements of tumor microenvironment in pathophysiologically relevant configurations. It is necessary to investigate the microenvironmental influence of brain to understand the unique characteristics of GBM invasion.

In a single-cell level, different modes of cancer cell invasion seem to exist, including mesenchymal and amoeboid type. The mode of cell invasion has been classified based on the morphology of invasion pattern, the molecular parameters, and the modification of ECM imposing by invading cells^14^. Unlike to the amoeboid mode, which is based on propulsive movement without proteolytic ECM remodeling, the mesenchymal mode involves focalized cell-ECM interactions and movement in a traction-dependent manner due to the cytoskeletal contractility. The focalized cell-ECM interaction leads to recruit ECM-degrading proteolytic enzymes to perform ECM remodeling and generate the path for invading cells^15^.

Numerous culture models, such as *in vitro* three-dimensional (3D) hydrogels, *ex vivo* organotypic brain slice cultures, and *in vivo* mouse models, have been used to study the cellular effects on microenvironment^11^,^16^,^17^,^18^,^19^,^20^,^21^,^22^,^23^,^24^. These research have indicated that the glioblastoma cells migrate individually with the mesenchymal mode of motility in a traction-dependent manner, so called saltatory migration^25^,^26^,^27^. In this process, the GBM cells generate a strong adhesion force at the focal contacts on ECM by intensively concentrating the integrins^25^. Simultaneously, they produce the proteases to degrade the local ECM components, by pulling and contracting the actin cytoskeleton to propel toward a newly developed invasion path^28^. For example, proteolytic enzymes including matrix metalloproteases (MMPs) are intensively involved in ECM remodeling at these focal sites^25^,^29^. For this reason, targeting the proteolytic process in mesenchymal movement of GBM cells has been highlighted as a promising therapy to inhibit wide invasion. However, treatment with MMP inhibitors (MMPIs) has limitations, which cause severe side effects including musculoskeletal pain and inflammation^30^. Furthermore, despite intensive efforts, there has been little improvement offered by the new therapeutic strategies.

In this context, the effects of the tumor microenvironment on GBM invasion must be understood as the unique and complex features of brain tumor are simultaneously considered. A biomimetic approach to construct an environment relevant to brain tumor would be valuable in understanding the brain tumor biology and developing new therapeutic intervention. We hypothesized that GBM has different strategies of invasion that are involved in adapting to its local microenvironment to facilitate invasion. To test this hypothesis, we developed an *in vitro* biomimetic microenvironment platform that mimics the complex microenvironment of GBM in physiologically relevant configuration.

## Results

### Abnormal regulation of hyaluronic acid (HA)-related genes in GBM tissue

**Figure 1:**
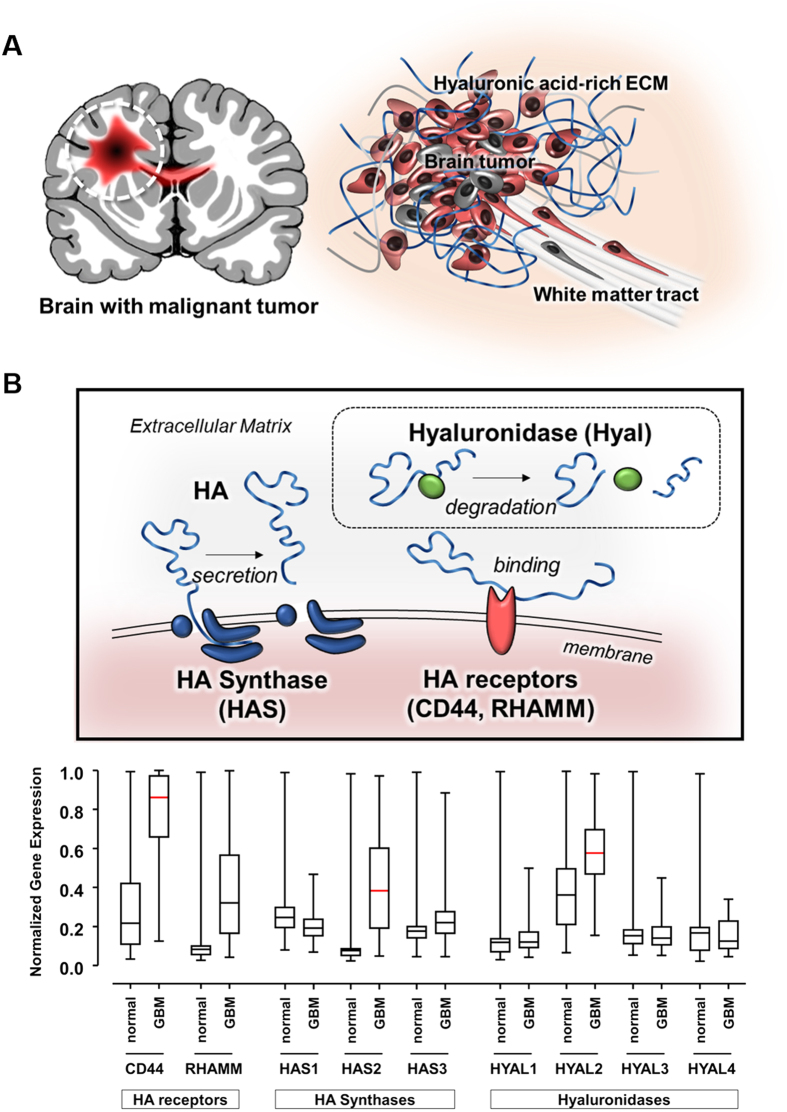
Pro-invasive factors within brain tumor microenvironment. (**A**) Schematic illustration of brain tumor-favorable microenvironment. Hyaluronic acid (HA)-rich extracellular matrix (ECM) and pre-existing brain anatomy (e.g. white matter tract). (**B**) Illustration of three major components in HA metabolism. Transcriptomic analysis of Gene Expression Omnibus (GEO) data for three major HA metabolic genes between normal brain tissue and GBM tissue: HA receptors (CD44, RHAMM), HA Synthases (HAS1, HAS2, and HAS3), and Hyaluronidase (HYAL1, HYAL2, HYAL3, and HYAL4). The normalized gene expression ranging from 0 (no expression) and 1 (fully expression). Red bar indicates the statistical significance between normal and GBM tissue.

There are two major components of microenvironment around GBM that could regulate GBM behavior (Fig. 1A). As biophysical cues, tumor cells interact with pre-existing brain anatomy (e.g. white matter tracts), called Scherer’s structures^13^, which are important as invasion path of GBM cells^26^. In addition, the ECM provides biochemical stimuli and mutually interacts with tumor cells. Although the components of the ECM in brain tumor tissue have not been fully elucidated yet, it has been reported that amorphous ECM at the invasive front of proliferating GBM is highly expressed such as glycosaminoglycans (GAGs), especially hyaluronic acid (HA)^31^,^32^,^33^, and they strongly up-regulated fibrous and adhesive ECM proteins, (e.g., collagen IV, fibronectin, and laminin)^34^.

To confirm the pathophysiologic gene regulation in GBM patients, we compared genomic expression of normal and GBM tumor tissue using the Gene Expression Omnibus (GEO) database. At the mRNA level, HA-related genes were abnormally regulated in GBM tumors, particularly those involved in HA metabolism (Fig. 1B)^35^. The encoded genes for HA-binding receptors, *CD44* and *RHAMM* (receptors for HA-mediated motility), were up-regulated in tumorous condition, possibly due to HA overexpression, contributing to GBM invasiveness^36^. Moreover, the encoded gene for HA synthase (HAS), especially *HAS2*, was aberrantly up-regulated in tumor, relative to *HAS* genes in normal tissue, which was consistent that *HAS* overexpression has been shown to result in increased tumorigenic features^37^. By contrast, in case of HA degradable genes, the expression of hyaluronidases (*HYAL*s), which are responsible for HA matrix catabolism, did not differ between normal and GBM samples, except *HYAL2*. However, in case of MMPs, the representative proteases *MMP2* and *MMP9* were highly increased in GBM tissue. In addition, we analyzed the focal adhesion (FA)-related genes, such as FA kinase (*FAK*) and vinculin, which are known to highly involve in providing the degrading sites during mesenchymal mode of GBM invasion. Whereas there was no change in *FAK* expression, vinculin expression was increased in GBM than in normal tissue (Fig. S1). These results indicated that the genes, particularly the up-regulated HA-related ones, were abnormally regulated in GBM tissue.

### Formation of HA-COL semi-IPN hydrogel to construct HA-rich ECM environment

**Figure 2:**
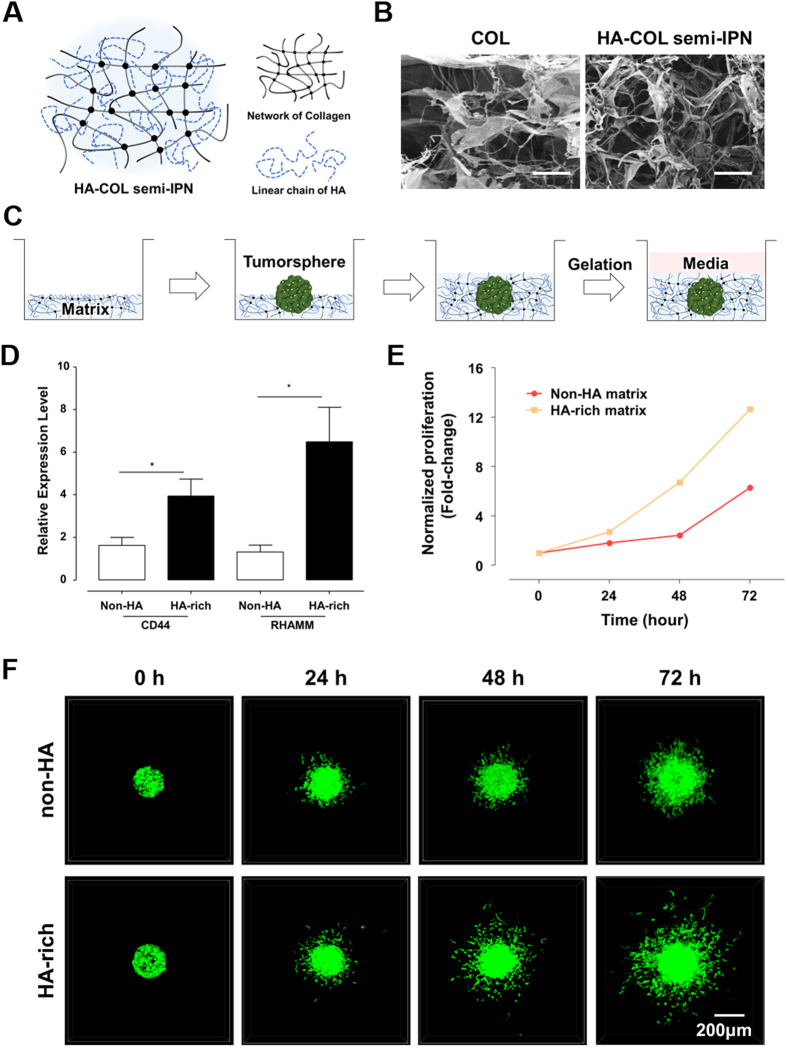
Features of GBM within Hyaluronic acid (HA)-rich ECM environment. HA can be interpenetrating with a fibrillary, crosslinked collagen (COL) hydrogel, forming hyaluronic acid-collagen semi-interpenetrating polymer network (HA-COL semi-IPN). The patient-derived GBM cells were isolated with CD133 markers using flow cytometry, then labelled with green fluorescent protein (GFP). (**A**) Structural scheme for HA-COL semi-IPN as HA-rich ECM environment.(**B**) Scanning electron images of COL and HA-COL semi-IPN matrices. Scale bar: 10 μm. (**C**) The culture process of three-dimensional GBM tumorsphere (TS) in ECM matrix. (**D**) Increased relative expression of HA receptors, CD44 and RHAMM in GBM, cultured within non-HA (COL only) and HA-rich (HA-COL semi-IPN) environment. (n = 5~6; Asterisks indicate a significant difference by student’s t-test, *p < 0.05). (**E**) Normalized proliferation of GBM cultured in non-HA (COL only) and HA-rich (HA-COL semi-IPN) environment (red line: non-HA, yellow line: HA-rich) in a fold-change. (**F**) Representative fluorescent images of invaded GBM TS within non-HA and HA-rich environment according to the time-variant. The invasiveness of GBM TS within HA-rich ECM environment was higher than one of GBM TS within non-HA environment.

To investigate the effects of HA on GBM identified in the transcriptomic analysis, we prepared collagen (COL) and HA-COL semi-interpenetrating polymer network (semi-IPN) hydrogels to represent non-HA normal and HA-rich conditions in brain tissue. Since COL hydrogel has fibrillar structures, it forms a well-organized interconnected network. However, a cross-linked network cannot be solely formed in HA hydrogel due to a linear and long chain of HA. Therefore, we created a HA-COL semi-IPN hydrogel in which long HA chains were interpenetrated into a cross-linked COL network (Fig. 2A).

Various concentrations of HA solution were combined with 4 mg/ml COL hydrogel to optimize the HA-COL semi-IPN hydrogel. The most stable HA-COL semi-IPN gel was formed when using the 4 mg/ml HA, while higher concentrations of HA resulted in unstable mechanical features (Fig. S2A). Using scanning electron microscopy (SEM), the microscopic networks of COL and HA-COL semi-IPN hydrogels were observed (Fig. 2B). COL hydrogel exhibited thin, networked fibrils, while HA-COL semi-IPN hydrogel showed thickend fibrils, but there were no significant differences of porosity between two hydrogels. The moduli of COL and HA-COL semi-IPN hydrogels were measured to confirm the mechanical compatibility with brain tissue. Using the frequency sweep mode for the rheological analysis, the storage moduli (G’) were nearly consistent along all frequency ranges, showing higher values than loss moduli (G”) at each frequency. This indicated the stable and uniform formation of internal cross-linked polymer networks for both COL and HA-COL semi-IPN hydrogels (Fig. S2B). In addition, the elastic moduli at 1 Hz of both hydrogel (COL: 78 Pa, HA-COL semi-IPN: 118 Pa; Fig. S2C) were comparable with that of brain tissue^38^.

To evaluate the structural stability of HA-COL semi-IPN hydrogel within liquid phase-solution (*i.e.* cell culture medium), we measured the amount of HA released from HA-COL semi-IPN during 72 hr cultivation using the carbazole reaction assay to detect the solubilized form of glucuronic acid, a major component of HA. The HA concentration was calculated based on the standard curve for HA using optical density (O.D.) (Fig. S3A). We collected and tested the medium every 24 hr, which showed no significant changes in O.D. over time (Fig. S3B). Moreover, HA-COL semi-IPN gel maintained their initial networked structures overtime without deformation in absence of cell-ECM interaction (Fig. S3C). These results confirmed that HA-COL semi-IPN was stable, despite high solubility of HA.

### Influence of HA-rich environment on GBM

We observed the influence of an HA-rich environment on GBM using COL and HA-COL semi-IPN hydrogel as a non-HA and HA-rich matrices respectively. As 3D tumor masses can reflect *in vivo* biological configurations (Fig. 2C), we cultured spontaneously formed 3D tumorspheres (TSs) from patient-derived CD133-positive GBM cells in non-HA and HA-rich matrices. Genomic expression of HA receptors, *CD44* and *RHAMM* were significantly higher in GBM TSs in HA-rich environment (Fig. 2D). Moreover, GBM TSs cultured in the HA-rich matrix proliferated more than those in the non-HA matrix (Fig. 2E). In addition, when we observed GBM invasion within both matrices in time-lapse, GBM TS showed more aggressive infiltration into the HA-rich matrix, than TS invasion within non-HA matrix (Fig. 2F, Supplementary videos 1, 2).

At a single-cell level, we observed marked morphological differences in scattered cells within non-HA and HA-rich matrices using confocal microscopy. In HA-rich matrix, single GBM cells were round with thin tails, whereas cells in non-HA matrix were elongated (Fig. S4A,B). In addition, it was revealed that cells in HA-rich matrix had significantly lower tail-to-body ratios (Fig. S4C). In a dynamic aspect, we observed that for given time (6 h), the single GBM cell moved faster in HA-rich matrix, compared to cell in HA-free matrix (Fig. S4D). These results suggested that the HA-COL semi-IPN represented HA-rich environment of brain tumor tissue, promoting aggressive progression and invasion.

### Influence of fibrous structures on GBM

**Figure 3:**
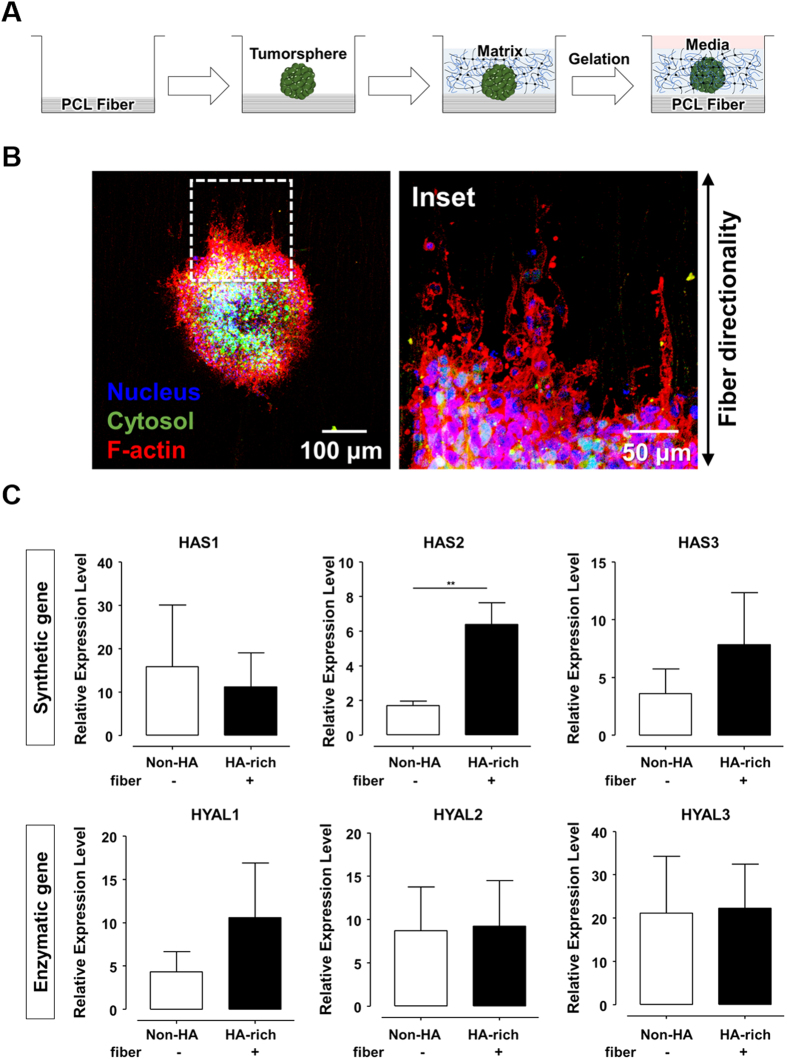
Features of GBM in *in vitro* biomimetic microenvironment platform. (**A**) The culture process of three-dimensional GBM tumorsphere (TS) in *in vitro* biomimetic microenvironment platform. (**B**) Morphological characteristics of GBM TSs within the *in vitro* biomimetic microenvironment platform. Fluorescent images of TSs along the electrospun fiber. (Blue: nucleus, Green: cytosol, Red: F-actin). (**C**) Relative expression of HA metabolic genes: synthetic genes (HAS) and enzymatic genes (HYAL) (n = 5~6; Asterisks indicate a significant difference by student’s t-test, **p < 0.001; no sign for Non-significant difference).

We examined whether biophysical cue, such as brain histopathology, could contribute to preferential infiltration at the invasive front in an HA-rich environment. To test this tissue interfacial invasion, we cultured GBM TSs in a submicron fiber-incorporated HA-COL semi-IPN matrix (Fig. 3A). Electrospun polycaprolactone (PCL) fibers, which mimic the representative Scherer’s structures, white matter tracts, had submicron-sized diameters (~3 μm) with cylindrical morphology (Fig. S5A), which could induce *in vivo*-like GBM cell movement based on our previous research^26^.

Using Fast Fourier Transform (FFT), we analyzed that the profiles of fibers were composed of symmetric central narrow bands, indicating nearly high alignment (Fig. S5B). A radial summation of normalized pixel intensities also confirmed nearly uniform fiber alignment, showing two dominant peaks at 90° and 270°, which represented symmetry (Fig. S5C).

F-actin was stained with rhodamine-conjugated phalloidin for observation. At the matrix-fiber interface, the F-actin of GBM cell was highly aligned and guided by the electrospun fibers, whereas the GBM TSs cultured without fibers exhibited randomized F-actin organization (Fig. 3B, Fig. S5D). In addition to the morphological changes, the fibrous structures influenced on the invasion dynamics of GBM TSs. At the invasive front, the scattered cells disseminated in the direction of the fibers (Fig. S5E). As a key factor in brain tumor microenvironment, we confirmed that the phenotypic characteristics of GBM TSs at the interfacial invasion front were largely dependent on the biophysical effects that mimic the pre-existing brain anatomic structures. Taken together, these results indicated PCL fibers-incorporated HA-COL semi-IPN hydrogel can be used as an effective *in vitro* biomimetic microenvironment platform.

### Features of GBM in an *in vitro* biomimetic microenvironment platform

We investigated gene regulation in GBM TSs using the biomimetic microenvironment platform, which is based on PCL fibers-incorporated HA-rich matrix. The expression profile was obtained from quantitative real-time (qRT) PCR analysis of GBM TSs cultured in the biomimetic platform (Fig. 3C, Fig. S6). As expected, intracellular signals related to HA metabolism were influenced by the presence of HA, showing the upregulation of *HAS*, especially *HAS2*. Conversely, there were no changes in *HYAL* expression. In particular, in contrast to the transcriptomic results, *HYAL2* showed no expressional change (Fig. 3C). The expression of *MMP* gene increased, whereas that of *FAK* was slightly downregulated in the platform (Fig. S6). These results were consistent with our transcriptomic analysis (see Fig. 1B). We concluded that GBM TSs cultured in our model were influenced by the biomimetic microenvironment, significantly regulating their ECM-related gene expression.

### *HAS* inhibition as a new therapeutic suggestion

HA had been proved to be not only a prominent component of the ECM in brain tumor microenvironment but also a driver of tumor malignancy^31^,^35^. Based on GEO analysis data, HA-related genes, especially *CD44, HAS2* and *HYAL2* were significantly upregulated in GBM tissue obtained from patients (see Fig. S1). These upregulated genes represent potential targets for brain tumor therapies. As *CD44* is a multifunctional molecule involved in cancer proliferation, differentiation, migration, or angiogenesis, there have been efforts to target the *CD44*. However, there have been few reports on targeting metabolic genes such as *HAS* and *HYAL* to develop therapeutic agents.

Recent research using animal models has found that inhibition of *HA* synthesis yields effective results in treating malignant diseases^39^. Therefore, we investigated the effect of HA synthesis inhibition on GBM invasion in our model. We found that 4-methylumbelliferone (4-MU), or hymecromone that is already used as a bile therapy, inhibits production of glucuronic acid (Fig. S7A), which is a major component of HA. In addition, 4-MU is an approved drug in Europe and Asia and has been identified as a potential anti-cancer drugs, including pancreatic, breast, and liver cancer.

**Figure 4:**
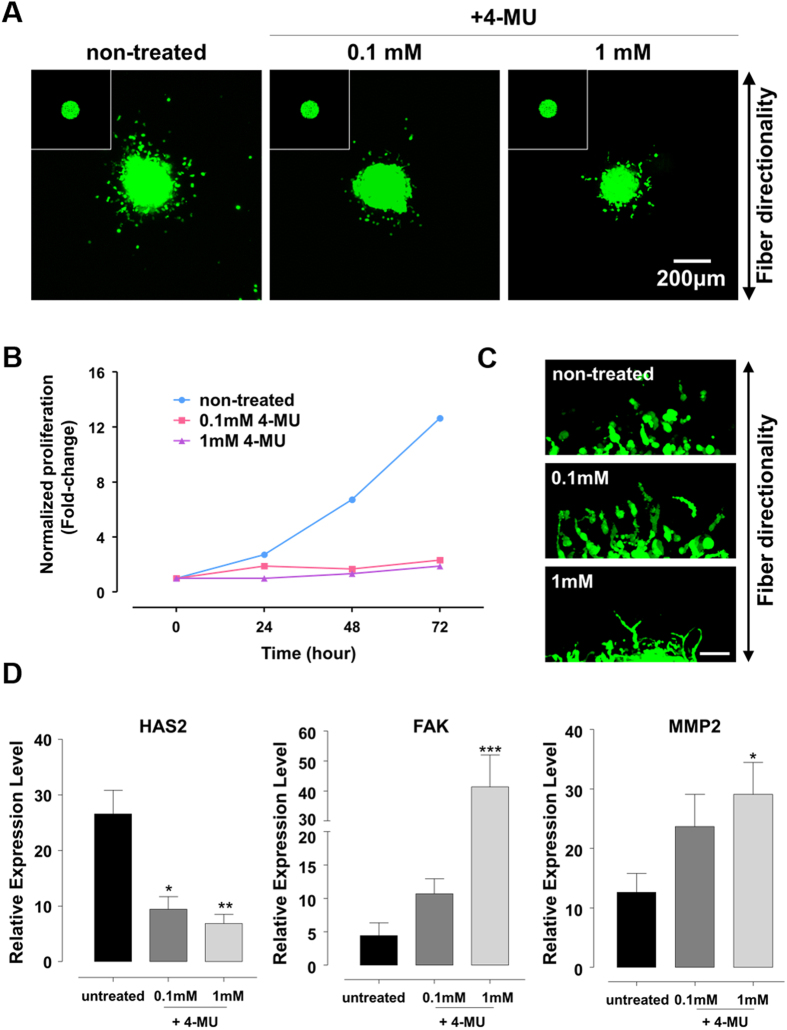
Inhibition of HAS as a new therapeutic suggestion. (**A**) The inhibition of HAS genes in 4-methylumbelliferone (4-MU) treated GBM TSs within biomimetic environment after 72 hr invasion. 4-MU was treated after 12 hr invasion. Inset: 4-MU treated GBM TSs at 0 h. (**B**) Normalized proliferation of GBM in presence of 4-MU treatment in a fold-change. (**A**) Magnified immunofluorescent images for invaded GBM TSs in presence of 4-MU treatment. The 4-MU was treated at 12-hour after GBM invasion. Scale bar: 50 μm. (**C**) Molecular expression profiles in presence of 4-MU. (n = 5~6; Asterisks indicate a significant difference statistically by student’s t-test, *p < 0.05; **p < 0.01; ***p < 0.001).

We observed the effect of HAS inhibition on TSs invasion for 72 hr by treating with 0, 0.1 and 1 mM of 4-MU (Fig. 4A). First, the GBM cells were allowed to invade for 12 hr and subsequently treated with 4-MU. In the HA-rich environment, 4-MU treated TSs showed low invasion compared with untreated TSs. Moreover, HAS inhibition significantly decreased TSs proliferation but not cell viability (Fig. 4B, Fig. S7B). When the TSs were treated with 4-MU at the beginning of the cell invasion (0 hr), little invasion also occurred in the presence of 4-MU (Fig. S8). In addition, to confirm the inhibition efficacy of 4-MU, we investigated the effect of HAS inhibition on GBM invasion in absence of HA. GBM TSs within PCL fiber-incorporated COL matrix were allowed to invade 12 hr and then treated 4-MU with 0, 0.1 and 1 mM concentration. There were no significant effect of the HAS inhibition on 4-MU treated TSs in HA-free matrix, compared to TSs in the HA-rich environment (Fig. S9A). Moreover, the inhibition of HA synthesis did not influence on TSs proliferation (Fig. S9B). These results collectively indicated that the 4-MU treatment was significantly effective in presence of HA.

Interestingly, with increasing 4-MU concentrations, the invaded GBM cells with sharper and thinner morphologies dominantly began to invade along the direction of fiber (Fig. 4C). These results were confirmed by molecular profiles of GBM TS in presence of 4-MU treatment (Fig. 4D). Correlated to HA inhibition by 4-MU, *HAS2* expression decreased in a dose-dependent manner. Consistent with morphological changes in GBM cells but in contrast to *HAS2* expression, *FAK* and *MMP2* expressions increased significantly and in proportional to 4-MU concentration. By contrast, in HA-free matrix, *HAS2* expression decreased in a dose-dependent manner, but *FAK* and *MMP2* expressions showed no marked changes in spite of 4-MU treatment (Fig. S9C). Thus, we revealed that in HA-rich environment, depletion of HA-dependent (especially *HAS2*) invasion activated other strategies mediated by FA with enzymatic process to adapt the microenvironment.

## Discussion

In this study, we hypothesized that GBM might alter their strategies of mesenchymal mode of invasion within their local microenvironment. To confirm this, we developed a 3D biomimetic GBM microenvironment platform that includes two major pro-invasive factors, a HA-rich ECM environment and the anatomical guidance of Scherer’s structure. The platform used in this study, enables to observe a modulation of invasion strategy of GBM through microenvironmental adaptation.

To investigate the GBM invasion in their microenvironmental context, we adopted HA-COL semi-IPN as HA-rich matrix and electrospun microfibers as brain anatomy-mimetic structures. In our platform, we could observe the phenotypic changes of GBM cells in response to the effect of HA-rich matrix, while being guided along a fibrous structure as interfacial invasion routes. Within the HA-rich matrix, the GBM cells were round, with low tail-to-body ratio, whereas cells were elongated along the fibrous structures, with high tail-to-body ratio, presenting distinct morphological difference in presence/absence of HA or fibers.

Next, we studied the microenvironmental influences on the motility of GBM TSs using our platform. It indicated that the motility of GBM TSs in HA-rich environment is faster than that of the TSs in HA-free matrix. According to the time-lapse observation, in HA-free and HA-rich matrices, the GBM TSs invaded in traction-mediated manner by developing and contracting thin, and finger-like structures, such as filopodia. Moreover, the GBM cells within HA-rich matrix experienced the rapid turnover of cytoskeletal reorganization during their movement. In case of GBM TSs invasion on the PCL fibers, the cells were locally influenced at the fiber-matrix interface, following the direction of fibers. GBM cells were simultaneously allowed to elongate their cytoskeletal structures along the fibers. These features observed in our platform were frequently reported by the histological samples from GBM patients^12^,^40^ with highly elongated morphology along pre-existing structures in the brain, such as white matter tracts and microvasculatures. Taken together, the distinctive morphologic and motile features of GBM TSs were reproduced in our platform.

Although phenotypes of GBM TSs were controlled by both biochemical (HA) and physical (fiber) cues, the molecular features of GBM cells in brain tumor-favorable environments were revealed to be mainly dependent on the biochemical stimuli from HA according to our results. The local invasion of GBM is reported to be highly associated with HA^32^. In addition, HA-binding proteins such as *CD44* and *RHAMM*, which mediate adhesion onto HA, are known to contribute to invasive motility during GBM progression^36^. In company with highly expressed HA and their HA-binding receptors, the metabolic process of HA is also regulated abnormally for both anabolic (HA synthesis) and catabolic (HA degradation) processes. According to our results, HA production by *HAS*, especially *HAS2* of GBM TSs was markedly activated during invasion into HA-rich ECM. Interestingly, the representative HA receptor, the *CD44* gene has a close relationship with *HAS*, suggesting that crosstalk between two genes might stimulate the signal cascades for GBM invasion^36^. Based on both the GEO database and experimental results, we confirmed that gene expression of HA receptors and *HAS* genes were up-regulated in tumorous conditions, suggesting the correlation with GBM infiltration. Therefore, we can suggest that GBM invasion is predominantly influenced by HA-rich ECM environments due to the high involvement of HA receptors and *HAS* in the infiltrating GBM.

Recently, anabolic HA genes, especially *HAS2*, have been identified as a potential therapeutic target due to their upregulation in tumorigenic conditions^41^,^42^, which was consistent with our analytical and experimental results. In response to HA increase, the synthetic process in HA metabolism presumably accelerated the positive feedback of HA production, contributing to HA-mediated tumorigenesis.

We thus suggested *HAS* gene as a new anti-invasion target. HA production was inhibited by 4-MU, which significantly lowered the invasive and progressive abilities of GBM. This inhibition was only effective in presence of HA through depletion of HA-dependent (especially *HAS2*) invasion, which was confirmed by the *HAS* inhibition of GBM TSs in absence of HA. However, we observed that GBM tried to find an alternative way for infiltration. Even after blocking the major invasion route mediated by *HAS*, the 4-MU treated GBM TSs showed slight invasion along the fibers at the matrix interface. Noticeably, when blocking *HAS* genes, the *FAK* genes, as well as the enzymatic *MMP2* genes of GBM TSs showed increased expression in proportion to 4-MU concentrations. This indicated that GBM shifted their invasion strategy from a *HAS2*-mediated route to a *FAK/MMP2*-mediated one through microenvironmental adaptation. These results signified the presence of various strategies in GBM invasion. Although the therapeutics prohibit the major routes of invasion mechanism in GBM, the GBM cells could find an alternative strategy, by adapting to other routes within the local microenvironment. Thus, it could be suggested that multi-target treatment is needed to block the various strategies of GBM invasion.

Meanwhile, the transcriptomic results suggested that *HYAL* genes, especially *HYAL2*, could be promising targets in treatment of GBM. However, the experimental results indicated no salient changes in *HYAL* expressions, and the role of *HYAL* in tumor tissue remains unknown. According to conflicting reports, *HYALs* can act as either tumor promoters or suppressors depending on concentration^41^,^43^. However, in the tumor tissue, tumor cell-derived *HYAL* acted mainly as a tumor promoter, so careful consideration is needed to target *HYAL* as a therapy.

In summary, we presented strategies of mesenchymal GBM invasion based on an *in vitro* biomimetic microenvironment platform. These results partially clarified an unconventional and poorly understood model of brain tumor invasion as well as the response of GBM to the external environment during invasion. In addition, we observed a microenvironmental adaptation in response to blockade of *HAS*-mediated invasion route. The GBM cells, which was blocked by 4-MU, can readily replace their invasion strategy in *FAK*/*MMP*-mediated manner, which might contribute to evasion or resistance to anti-cancer treatments. Therefore, multi-target therapies might be promising and successful in treating infiltrative GBM.

The microenvironment of brain tumor is more complex than we presented. GBM invasion plasticity is determined by intra-tumor heterogeneity and tumor-stromal reciprocity. Although our model might not have reflected all relevant conditions, we were able to target the major evading mechanisms of anti-invasion therapies using a biomimetic microenvironment platform for GBM cells. For tumor subpopulations with tumorigenic capacities and therapeutic resistance, future studies should focus on identifying new critical pro-invasive factors in brain tumors to determine other strategies of invasion.

## Materials and Methods

### Transcriptomic analysis of GEO data

The publicly available Gene Expression Omnibus (GEO) microarray database was utilized to provide the expression profiles of the genes of interest. The GEO microarray data is based on GPL570 Affymetric GeneChip Human Genome U133 Plus 2.0 array. The Oncopression analysis tool (www.oncopression.com) was used to retrieve mRNA expression data for genes of interest from all available GBM tumor samples, including HA receptors (*CD44, RHAMM*), HA synthases (*HAS1, HAS2, and HAS3*), hyaluronidases (*HYAL1, HYAL2, HYAL3*, and *HYAL4*), matrix metalloproteases (*MMP2* and *MMP9*) and focal adhesion (FA)-related molecules (*FAK* and *vinculin*). The gene expression level is normalized by Universal exPression Codes (UPC), providing the relative values from 0 (no expression) to 1 (fully expression).

### Preparation of collagen-hyaluronic acid semi-interpenetrating polymer network gels

We compare the COL and HA-COL semi-IPN hydrogel to represent normal (non-HA) and tumorous condition, respectively. For COL hydrogel as normal condition, COL solutions were prepared on ice immediately prior to use by diluting high-concentrated stock of COL type I from rat tail (Corning, USA) according to manufacturer’s protocol. Briefly, 8.23 mg/ml COL stock was diluted into 4.0 mg/ml collagen solution in distilled water. 1 M NaOH was added to bring the pH to 7.4 for cell culture. To fabricate of HA-COL semi-IPN, the solution of sodium hyaluronate (Lifecore Biomedical, USA, 1.01~1.8 MDa) with concentration of 4, 8 and 16 mg/ml were used for diluting solvent, instead of distilled water in the process of preparation^44^. Solutions were mixed thoroughly prior to hydrogel gelation into cell culture platform or rheology dishes. Both hydrogels were incubated at 37 °C for 1 h prior to addition of a superlayer of cell culture medium. For further inspection, the cross-sections of freeze-dried hydrogels were coated with platinum (Pt) and observed with conventional scanning electron microscopy (SEM, FEI, Netherlands).

### Fabrication of electrospun PCL fiber

For mimicking the pre-existing structure of white matter tract, electrospinning technique was used to fabricate aligned fiber in sub-micron using biodegradable 15% polycaprolactone (PCL) polymer (Sigma-Aldrich, St Louis, MO, 440752). 15% PCL polymer were firstly melted in chloroform/N,N-dimethylformamide solvent (3:1 v/v ratio), purchased in Sigma-Aldrich (372978 and 227056, respectively). Using a syringe with 25-gauge blunt metal needle, the solubilized PCL polymer was jetted into the coverglass on the rotating mandrel (2500 rpm) at the 13kV of voltage, with the speed of 1.0 mL/h. The air gap between the needle and collector was set to 15 cm. The collected fibers were sterilized with PBS for 24h with ultraviolet rays for further experiment. Platinum (Pt)-coated electrospun fiber sample for conventional scanning electron microscopy (SEM, FEI, Netherlands) were prepared for visual inspection.

### Cell culture

The patient-derived GBM stem cell line, GSC11 used in this study was kindly provided by Dr. F. Lang (Department of Neurosurgery, The University of Texas, MD Anderson Cancer Center, TX, USA). GSC11 was CD133-positive GBM subpopulation sorted by flow cytometry and established as GBM stem cell line to spontaneously form the GBM tumorspheres (TSs)^45^,^46^. The TSs were transfected with green fluorescent protein (GFP) for easy visualization. The TSs were cultured in Dulbecco’s modified Eagle medium: nutrient mixture F-12 (DMEM/F12) (Welgene, Korea) supplemented with 1% penicillin/streptomycin (Welgene), 1× B27 (Invitrogen, Carlsbad, CA, USA), 20ng/ml of basic fibroblast growth factor (bFGF, Invitrogen) and 20ng/ml of epidermal growth factor (EGF, Invitrogen). The cells were maintained at 37 °C in an atmosphere of 5% CO2 and 95% air. A confluent condition of TSs was passaged every 5 days (1:5 ratio) by dissociating with accutase (Invitrogen). A sphere around 10,000 cells were embedded within the platform for further experiment.

### Invasion assay

For evaluating the invasive ability, the GFP-tagged TSs were embedded within 3D *in vitro* platform for 4 days. The visual inspection was performed using confocal microscope (Nikon, Japan), by scanning the whole depth of invaded TSs. The presented images were projected in the direction of depth (z-axis). The signal of GFP from TSs were recorded to quantify the invaded area by calculating the number of pixels. For further investigation for inhibition of invasion, the 4-methylumbelliferone (4-MU, Abcam, Cambridge, U.K.), so called hymecromone, is used to block the HA synthesis through the HAS.

### Rheological analysis of hydrogel

To characterize the mechanical properties of COL and HA-COL semi-IPN hydrogels, the rheological features of hydrogels were analyzed with a rotating rheometer (Bohlin Advanced Rheometer, Malvern Instruments, Worcestershire, U.K.). After gelation, both hydrogels were placed between a 20 mm rotational detector and a parallel plate of the rheometer. The rheological features of hydrogels were characterized in a frequency sweep mode with a controlled stress. The storage modulus (G′) and loss modulus (G″) of the hydrogel samples were recorded in the frequency sweep mode at a given 1% strain within predetermined frequency ranges (0.079−6.33 Hz). The elastic moduli for hydrogels were calculated at 1 Hz.

### HA-releasing assay

To confirm the solubility of HA-COL semi-IPN hydrogel within liquid phase-solution, we measured the solubilized form of glucuronic acid, one of HA component, using conventional carbazole reaction assay^47^. Briefly, we cultured GBM TSs within the HA-COL semi-IPN hydrogel and detected the released HA component in cell culture medium from the hydrogel during 72-hour incubation. At every 24-hour, we took the supernatant from the cell culture medium as experimental samples. A serial dilutions of standard or samples of 50 ml (1 mg/ml) were placed in 1.5 ml Eppendorf tubes. 200 ml of a solution of 25 mM sodium tetraborate (Sigma-Aldrich) in sulfuric acid (Sigma-Aldrich) was added. The tubes were heated for 10 min at 100 °C in an oven. After cooling at room temperature for 15 min, 50 ml of 0.125% carbazole (Sigma-Aldrich) in absolute ethanol were carefully added. After heating at 100 °C for 10 min in an oven and cooling at room temperature for 15 min, each sample was placed in 96-well plate to read in a microplate reader (Bio-Rad, Model 550) at a wavelength of 550 nm.

### Fast Fourier Transform (FFT) analysis of electrospun fiber

For quantified characterization of electrospun fiber, the Fast Fourier transform (FFT) analysis were used. The NIH ImageJ software supported by an oval profile plug-in was used to conduct 2D FFT analysis. The FFT analysis converts spatial information of image data into a mathematically defined frequency domain. This frequency domain plots the degree of pixel intensities in the spatial domain. The pixel intensities are summed along the peripheral direction for each angle of the circular projection from 0 to 360°. The summed pixel intensities for each radius are then plotted against the corresponding angle of acquisition to produce a graph of angle to arbitrary pixel intensity.

### Immunocytochemistry

TSs grown on 3D *in vitro* biomimetic microenvironment platform were fixed in 4% paraformaldehyde (Sigma-Aldrich) and permeabilized with 0.15% Triton X-100 (Sigma-Aldrich) for 30 minutes, respectively. F-actin was stained for 30 minutes using 2 mg/L rhodamine-conjugated phalloidin (Invitrogen). Nucleus was stained for 15 minutes using 2 mg/L DAPI (Invitrogen). After washing with PBS, the samples were preserved with Prolong Gold aqueous mounting medium (Invitrogen) before inspection by confocal microscopy (Nikon, Tokyo, Japan).

### Quantitative Real-Time Polymerase Chain Reaction (qRT-PCR)

After 7 days, the gene expression of fully-invaded TSs within biomimetic environment was examined with qRT-PCR. Total RNA was extracted from each sample (at least n = 4~6 per each group) according to conventional RNA extraction protocol. Briefly, the samples were prepared with RNA isolation reagent and extracted with chloroform after 10 min, 14000 rpm centrifugation. Reverse transcription was carried out using the cDNA Synthesis kit (Bio-rad, Richmond, CA, USA). qRT-PCR was performed using a PCR System (Bio-rad, Richmond, CA, USA) with PCR Master Mix (Toyobo, Japan). Gene expression of each marker was quantified using signal amplification of SYBR green (Bio-rad) for human CD44, human RHAMM, human hyaluronidase 1, 2 and 3, human hyaluronan synthase 1, 2 and 3, human matrix metalloproteinase 2, 9 and human glyceraldehyde-3-phosphate dehydrogenase (GAPDH). The level of gene expression was determined with the comparative Ct method in which the target genes were normalized to the endogeneous reference (GAPDH). The forward/reverse sequences used for experiments was listed in Table 1.

### WST-1 assay

We utilized the conventional WST-1 method with EZ-cytox assay (water-soluble tetrazolium salt method) to evaluate the cell proliferation and viability. Briefly, TSs were incubated with 10 μl EZ-cytox solution for 3-hour. After the formation of formazan crystals, by confirming color change of the culture supernatant, the absorbance of the culture supernatant was measured using a microplate reader at 450 nm (Bio-Rad, Richmond, CA, USA). The proliferation and viability rates were measured using the value of optical density (O.D.), compared to non-reacted EZ-cytox solution. The value of O.D. was relatively calculated as a normalized fold-change along the time-variant.

### Statistical analysis

Data are presented as the mean ± standard error of the mean (SEM). Levels of significance for comparisons between two independent samples were determined using the Student’s t-test. Groups were compared by one-way analysis of variance (ANOVA) with Tukey’s post-hoc test applied to significant main effects.

## Additional Information

**How to cite this article**: Cha, J. *et al*. Strategies of Mesenchymal Invasion of Patient-derived Brain Tumors: Microenvironmental Adaptation. *Sci. Rep.* **6**, 24912; doi: 10.1038/srep24912 (2016).

## Supplementary Material

Supplementary Information

Supplementary Video 1

Supplementary Video 2
