# Supplementary material for: Strategies of Mesenchymal Invasion of Patient-derived Brain Tumors: Microenvironmental Adaptation
Source: Sci Rep. 2016 Apr 25;6:24912. doi: 10.1038/srep24912 (PMC4842976; doi:10.1038/srep24912)
Supplement: Supplementary Information [file srep24912-s1.pdf]

Supporting Information

Strategies of Mesenchymal Invasion of Patient-derived Brain Tumors: Microenvironmental Adaptation

Junghwa Cha[a], Seok-Gu Kang[b] and Pilnam Kim[a,*]

[a]Department of Bio and Brain Engineering, KAIST, Daejeon 34141, Korea

[b]Department of Neurosurgery, Severance Hospital, Yonsei University College of Medicine, Seoul 03722, Korea

*To whom correspondence should be addressed. Email: pkim@kaist.ac.kr

Figure S1

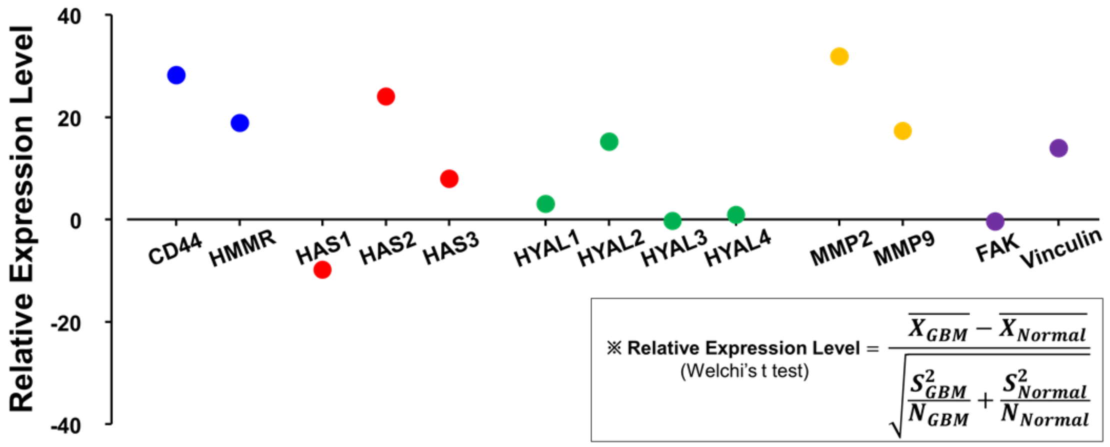

Gene	HA receptors		HA synthases			Hyaluronidases				Proteolytic enzymes		Adhesion molecules	
	CD44	RHAMM	HAS1	HAS2	HAS3	HYAL1	HYAL2	HYAL3	HYAL4	MMP2	MMP9	FAK	Vinculin
Welchi's t	28.22	18.87	-9.81	24.05	7.96	3.08	15.25	-0.25	0.922	31.89	17.35	-0.36	13.97

Figure S1.

Statistical analysis with Welchi's t test to evaluate the relative expression levels in the genes of interest. Gene expression data extracted from Genome Expression Omnibus (GEO) database. HA receptors (CD44, RHAMM), HA Synthases (HAS1, HAS2, and HAS3), and Hyaluronidase (HYAL1, HYAL2, HYAL3, and HYAL4) were investigated. The high value of relative expression level from Welchi's t test, the more significant difference in gene expression between normal and GBM tissue.

Figure S2

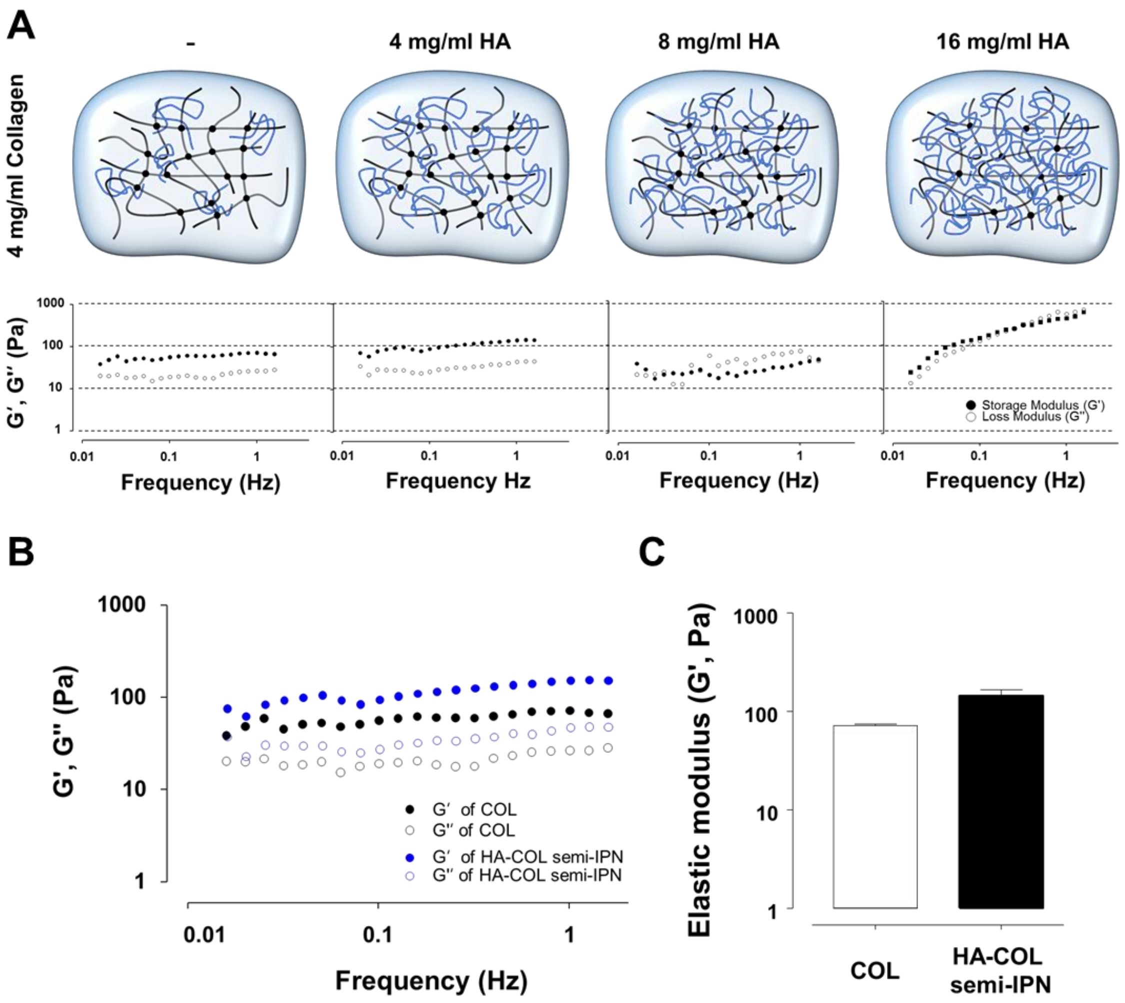

Figure S2.

(A) Rheological properties of non-HA (COL) and HA-rich (HA-COL semi-IPN) hydrogel. Storage modulus (G') and loss modulus (G') were recorded in a frequency sweep mode.

(B) Mechanical properties of non-HA (COL only) and HA-rich (HA-COL semi-IPN) hydrogel. Storage modulus (G') and loss modulus (G') were recorded in a frequency sweep mode.

(C) Average elastic modulus of control and HA-rich matrix at a frequency of 1 Hz (n=4).

Figure S3

A

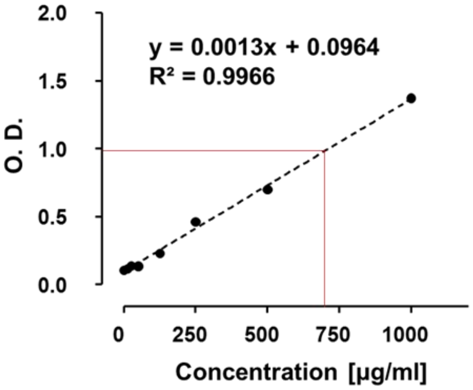

μg/ml	O.D
1000	1.37025
500	0.6975
250	0.460375
125	0.230375
50	0.13625
25	0.138
12.5	0.116875
0	0.10575

B

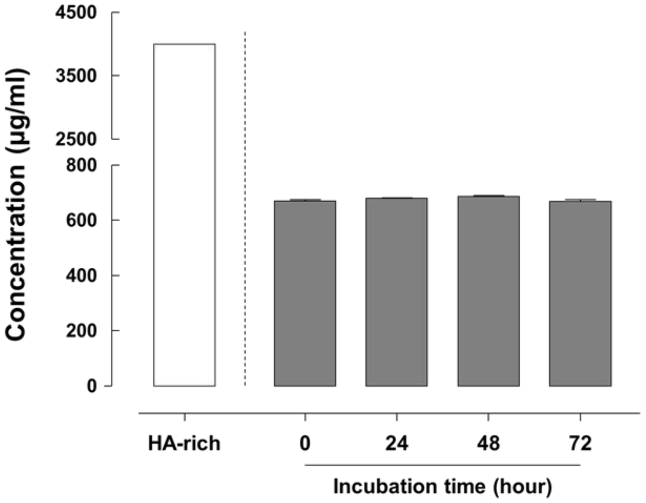

Time (h)	O.D	μg/ml
0	0.9670	669.692
24	0.9795	679.308
48	0.9891	686.664
72	0.9649	668.106

C

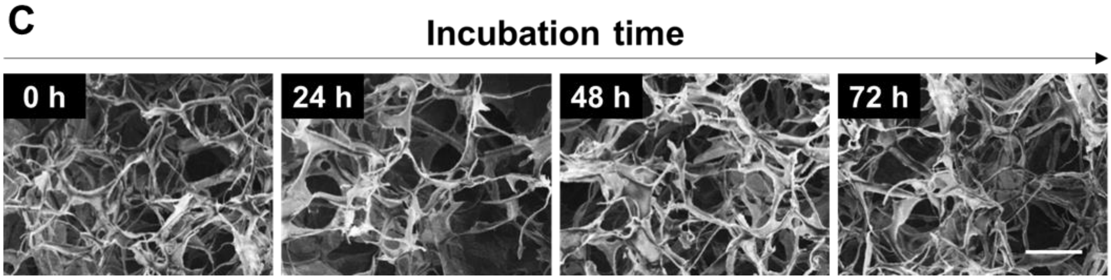

Figure S3.

HA content was analyzed by carbazole reaction assay to figure out the HA-COL semi-IPN stability in liquid phase.

(A) The standard curve for HA solution.

(B) Changes of HA concentration released from the incubated culture medium. When comparing the HA concentration with HA-rich ECM matrix, the concentration showed no significant difference along the time-variant (n=4 for each group).

(C) Scanning electron images of HA-COL semi-IPN matrices according to incubation time. Scale bar: 10 μm.

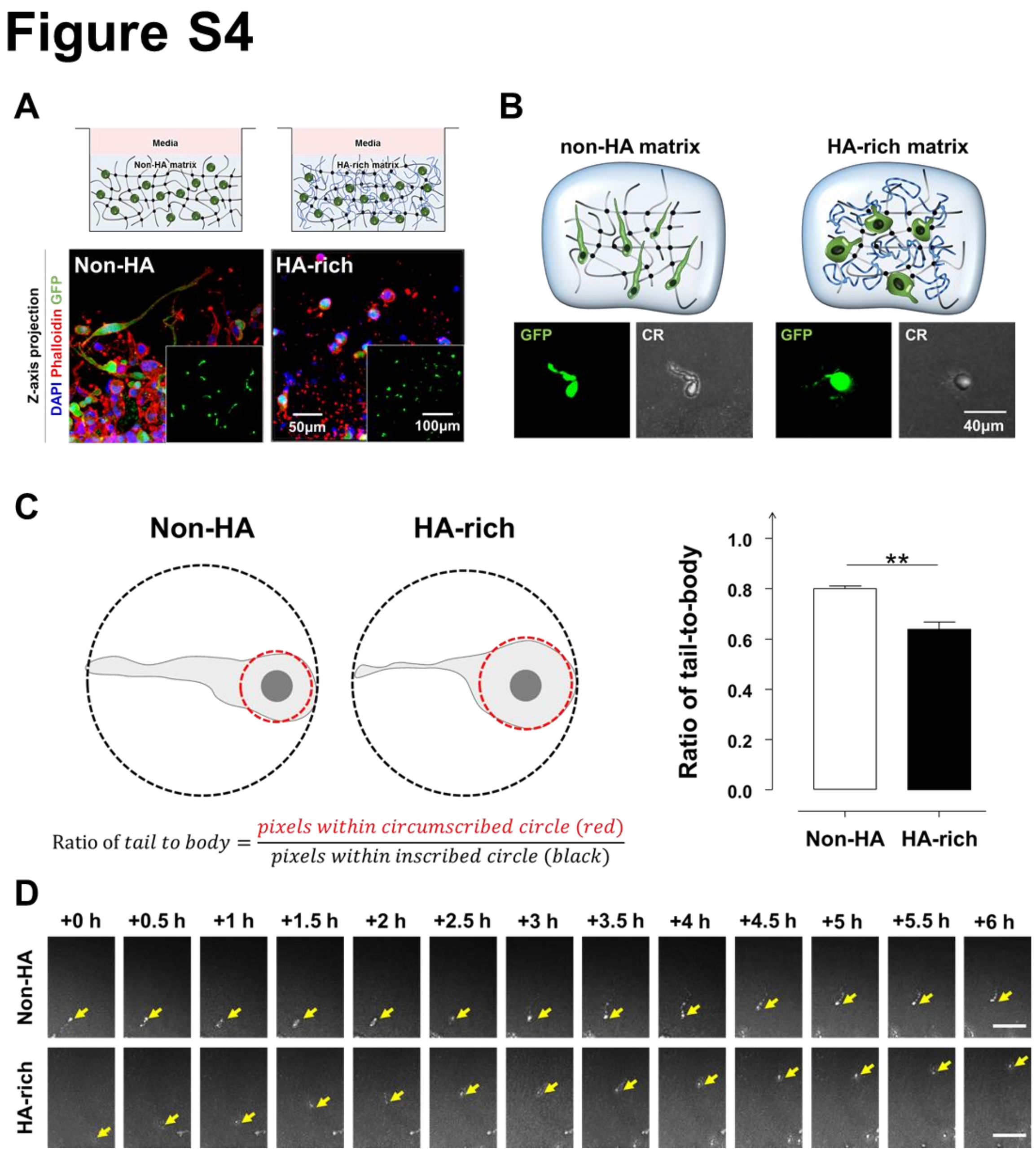

Figure S4.

(A) The morphological features of single-celled GBM within non-HA (COL only) and HA-rich (HA-COL semi-IPN) environment. The three-dimensional (3D) confocal images were projected into the depth (z)-direction (z-projection).

(B) Morphological features of GBM in non-HA (COL only) and HA-rich (HA-COL semi-IPN) environment. The images was obtained using both fluorescent and confocal reflectance (CR) mode

in confocal microscope.

(C) The ratio of tail-to-body for evaluation of single-celled GBM. (n=10~15 for each group; Asterisks indicate a significant difference by student's t-test, ** $p < 0.01$; no sing for Non-significant difference.)

(D) Time-lapse observation of single GBM cell in HA-free and HA-rich matrices. The images were taken every 0.5 hour. Scale bar: 50 μm.

Figure S5

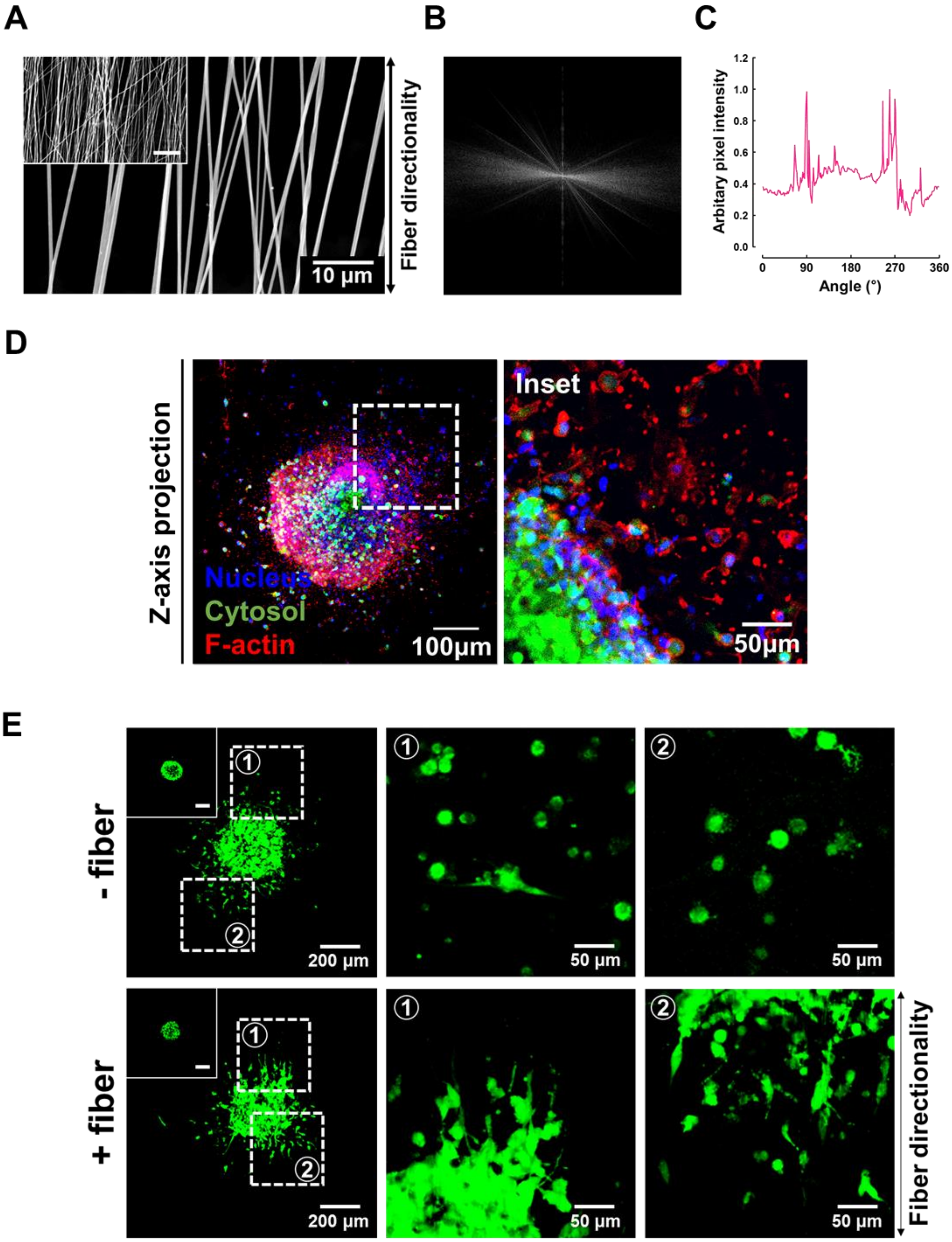

Figure S5.

(A) SEM images for electrospun PCL fiber to mimic the brain anatomy.

(B) FFT analysis of PCL fiber with FFT analysis.

(C) A radial summation of pixel intensities of PCL fibers

(D) Morphological characteristics of GBM TSs within only HA-rich ECM environment. Fluorescent images of GBM TSs with magnified images (inset). (Blue: nucleus, Green: cytosol, Red: F-actin)

(E) Features of invading GBM cells within HA-rich ECM in absence/presence of electrospun fibers. The disseminated cells at the invasive front of GBM TS were guided along the directionality in presence of fiber. (Inset: the GBM TS at the initial time of invasion)

Figure S6

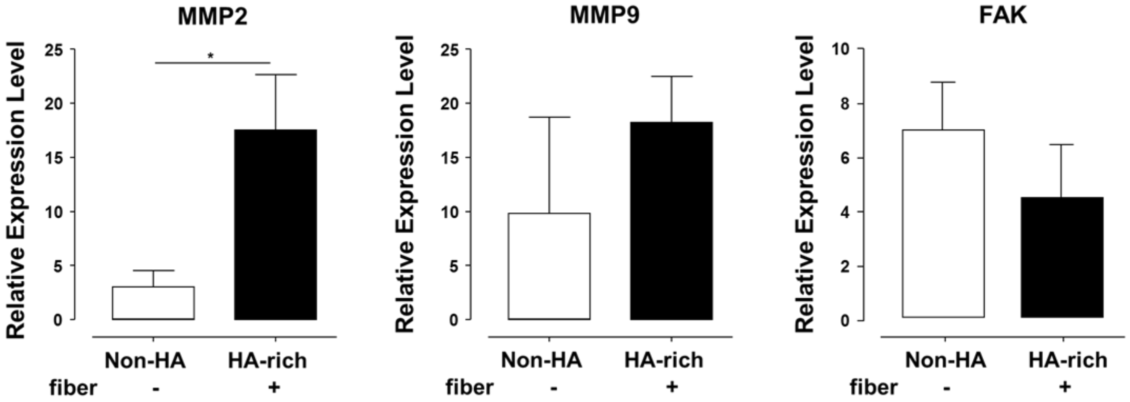

Figure S6.

Molecular expression profiles of proteolytic enzymes (*MMP2* and *MMP9*) and adhesion molecules (*FAK*) in GBM. (n=5~6; Asterisks indicate a significant difference by student's t-test, * $p < 0.05$; no sign for Non-significant difference.)

Figure S7

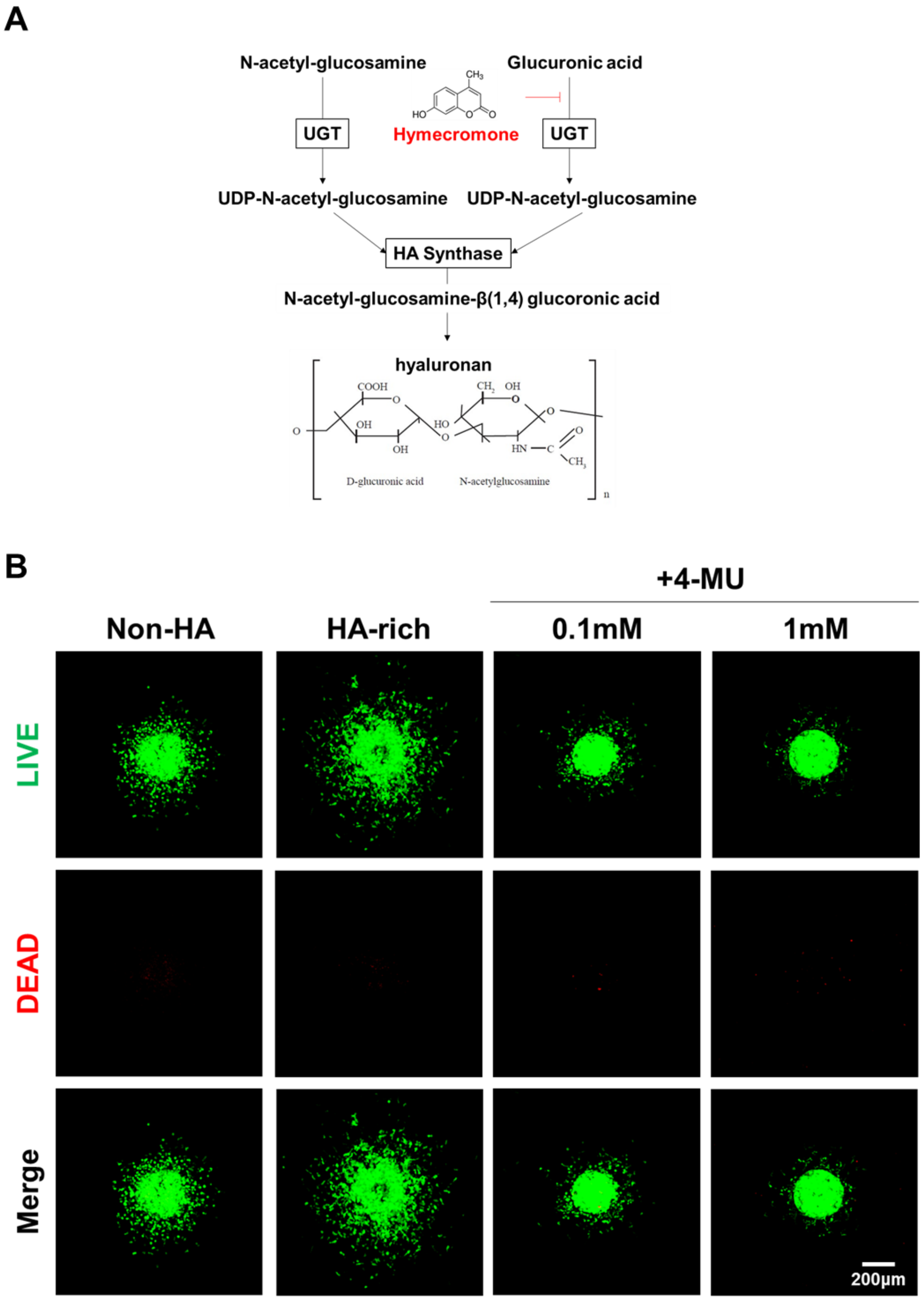
A
N-acetyl-glucosamine
Glucuronic acid
UGT
Hymecromone
UGT
UDP-N-acetyl-glucosamine
UDP-N-acetyl-glucosamine
HA Synthase
N-acetyl-glucosamine-β(1,4) glucoronic acid
hyaluronan
D-glucuronic acid
N-acetylglucosamine
B
+4-MU
Non-HA
HA-rich
0.1mM
1mM
LIVE
DEAD
Merge
200μm

Figure S7.

(A) Mechanism of action for hyaluronic acid synthase (HAS) inhibitor, 4-methylumbelliferone.

(B) Cell viability test with LIVE/DEAD staining kit for GBM TSs after 12 hr invasion, when treated with 0, 0.1, and 1mM 4-MU.

Figure S8

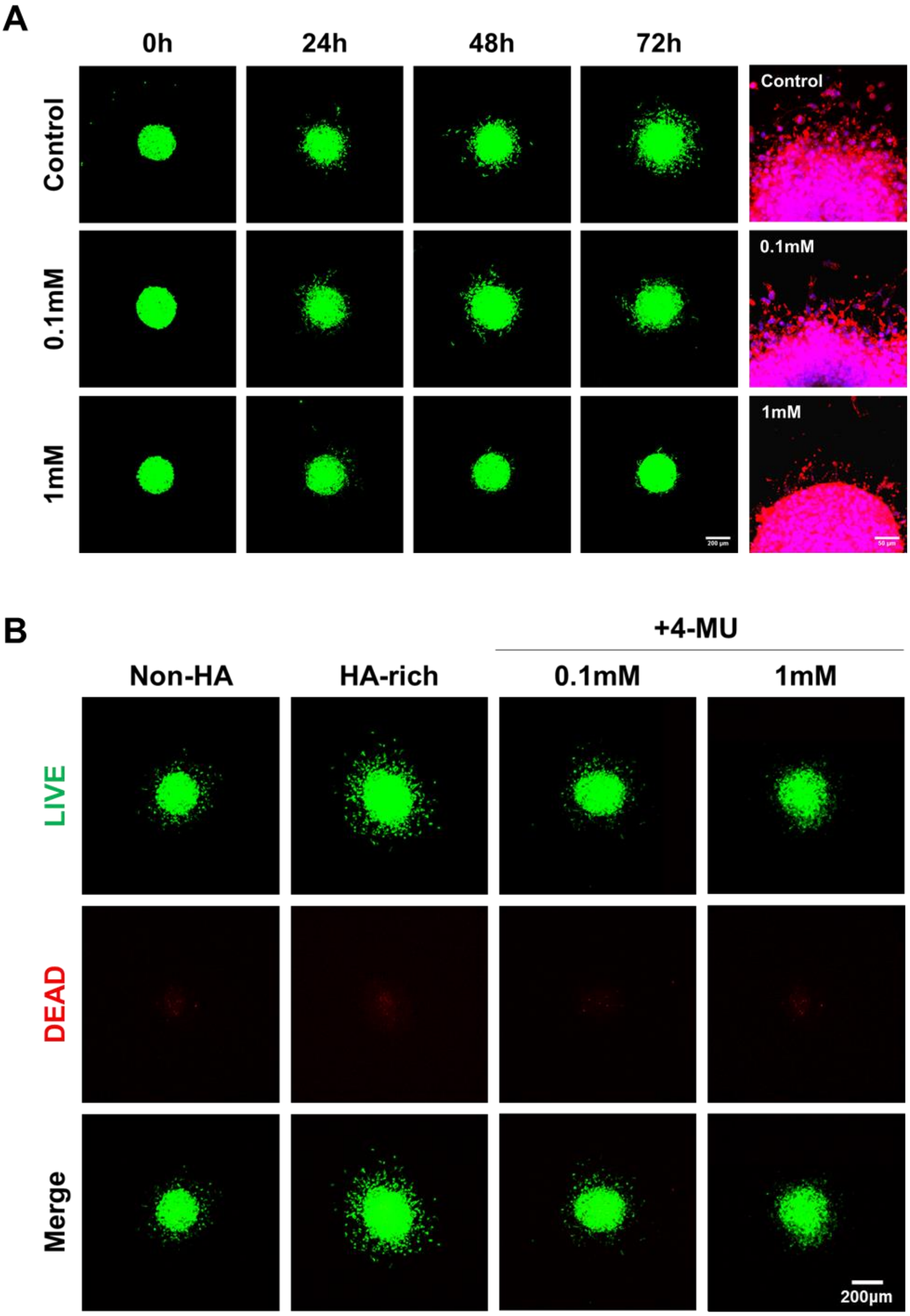
A
0h
24h
48h
72h
Control
0.1mM
1mM
Control
0.1mM
1mM
B
+4-MU
Non-HA
HA-rich
0.1mM
1mM
LIVE
DEAD
Merge
200μm

Figure S8.

(A) Comparison of morphology and invasion pattern for GBM TSs, right after (0 hr) treated with 0, 0.1, and 1mM 4-MU.

(B) Cell viability test when the 4-MU was treated right after (0 hr) GBM invasion. No significant effect on cell viability at the concentration of 0.1 and 1mM 4-MU.

Figure S9

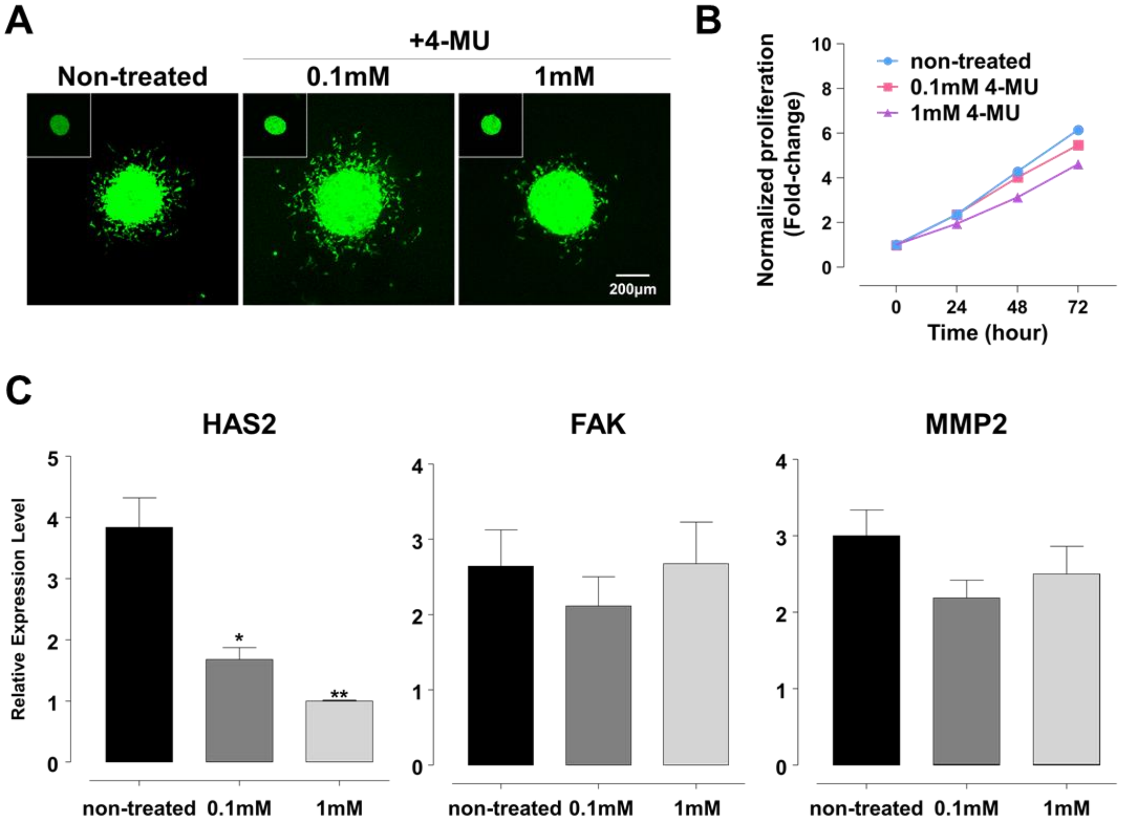

Figure S9.

(A) The inhibition of HAS genes in GBM TSs within PCL fiber-incorporated COL-based hydrogel platform using 4-MU after 12 hr invasion. Inset: 4-MU treated GBM TSs at 0 hr.

(B) Normalized proliferation of GBM in presence of 4-MU treatment in a fold-change.

(C) Molecular expression profiles in presence of 4-MU. (n=5~6; Asterisks indicate a significant difference statistically by student's t-test, * $p < 0.05$; ** $p < 0.01$; ***$p < 0.001$)

Table 1

Gene	Forward primer (5’ → 3’)	Reverse primer (3’ → 5’)
hGAPDH	GTATGACAACAGCCTTCAAGAT	AGTCCTTCCACGATACCAAA
hCD44	CTCTCTCCCTCCACTTCAC	GCCTAATGTCCAGTTTCTTTCA
hRHAMM	GTTTCTGGAGCTGCTCCGTC	ACTGGTCCTTTCAATACTTCTAAAGT
hHAS1	ATGCAGGATACACAGTGGAAGTAG	GGTGGGGACGTGCGGATC
hHAS2	TCCAACCATGGGATCTTCTT	GTGGATTATGTACAGGTTTCTGA
hHAS3	CTCTACTCCCTCCTCTATATGTC	AACTGCCACCCAGATGGA
hHYAL1	CAGGGTTAAGGAGGAGGA	CATCAAGGAGTATATGGACACT
hHYAL2	GATTACCTGACACGGCTG	GAAACTGTTGGTGCTGAGA
hHYAL3	TTTCCCTGCTGCCACTT	CTTGGGAGGGTTGACTGTAA
hMMP2	GACGGTAAGGACGGACTC	ACTTCACACGGACCACTT
hMMP9	GCCCAGCCCACCTCCACTCCTC	TGGGCTACGTGACCTATGACAT
hFAK	CCAGGAGAGAATGAAGCAAA	CAACAAACTAAAGGGAGGGTAT

Table 1. Primer Sequences Used in RT-PCR
